# Fertility-Sparing Treatment for Endometrial Cancer or Atypical Endometrial Hyperplasia Patients With Obesity

**DOI:** 10.3389/fonc.2022.812346

**Published:** 2022-02-18

**Authors:** Junyu Chen, Dongyan Cao, Jiaxin Yang, Mei Yu, Huimei Zhou, Ninghai Cheng, Jinhui Wang, Ying Zhang, Peng Peng, Keng Shen

**Affiliations:** Department of Obstetrics and Gynecology, National Clinical Research Center for Obstetric & Gynecologic Diseases, Peking Union Medical College Hospital, Chinese Academy of Medical Sciences & Peking Union Medical College, Beijing, China

**Keywords:** endometrial cancer, atypical endometrial hyperplasia, obesity, fertility-sparing treatment, GnRHa, progestin

## Abstract

**Objective:**

To evaluate the efficacy and prognosis of fertility-sparing treatment on endometrial cancer (EC) and atypical endometrial hyperplasia (AEH) patients with BMI ≥ 30 kg/m^2^.

**Methods:**

A total of 102 EC or AEH patients with obesity who received fertility-preserving therapy in the Department of Obstetrics and Gynecology, Peking Union Medical College Hospital were included in our study. All patients were followed up regularly. Clinical characteristics, treatment outcomes, adverse events, and reproductive outcomes were collected and analyzed.

**Results:**

A total of 88 (86.3%) patients achieved complete response (CR), 92.5% in AEH and 82.3% in EC, with 6 months (3–12 months) median CR time. High remission rates were found in patients who received gonadotropin-releasing hormone agonist (GnRHa)-based regimen, were younger than 35 years old, and lost more than 10% of their weight. Fifteen (17.0%) women had developed recurrence with a median recurrence time of 26 (8–52) months. Patients who received GnRHa regimen, lost more than 10% weight, received maintenance therapy, or conceived during the follow-up period had a low probability of recurrence. Of the patients with CR, 57 women attempted to get pregnant and 16 (28.1%) patients became pregnant, 7 (12.3%) of them successfully delivered and 4 (7.0%) were in pregnancy, while 5 (8.8%) of them miscarried.

**Conclusion:**

For obese patients with EC and AEH, fertility-preserving treatment can still achieve a promising response. Weight loss of more than 10% has a positive influence on response, recurrence, as well as pregnancy rates. GnRHa could be an option for obese women due to less effect on weight gain compared to progestin therapy.

## Introduction

Endometrial cancer (EC) is one of the most common and an increasingly problematic gynecological cancer, whose incidence has gradually risen in recent years (1). With the significant increase in the proportion of the obese population, the number of premenopausal EC patients of childbearing age increased (2). The standard treatment requires, at a minimum, a hysterectomy with bilateral salpingo-oophorectomy, and pelvic and para-aortic lymphadenectomy, if indicated. However, this standard treatment results in a permanent loss of fertility while young patients have a strong desire to bear children. Therefore, fertility-sparing treatment should be discussed in young patients who wish to preserve their fertility. To date, conservative management for young patients has been applied and showed encouraging results on treatment and reproductive outcomes (3–5). Factors such as obesity might be associated with the oncologic and reproductive outcomes (6). Many researchers have shown that weight gain increases the risk of developing endometrial cancer (7). The risk of endometrial cancer increases by 6 times when the body mass index (BMI) is over 40 kg/m^2^, and morbid obesity was associated with higher mortality and disease recurrence (8). Also, weight loss in obese women was associated with lower EC risk (9). Although EC is the cancer most strongly associated with obesity (10, 11), data on the effective application of conservative treatment for obese patients were relatively limited. Therefore, this study aimed to investigate the efficacy and safety of fertility-preserving therapy in obese EC or AEH patients.

## Methods

### Patients Recruited

**Figure 1:**
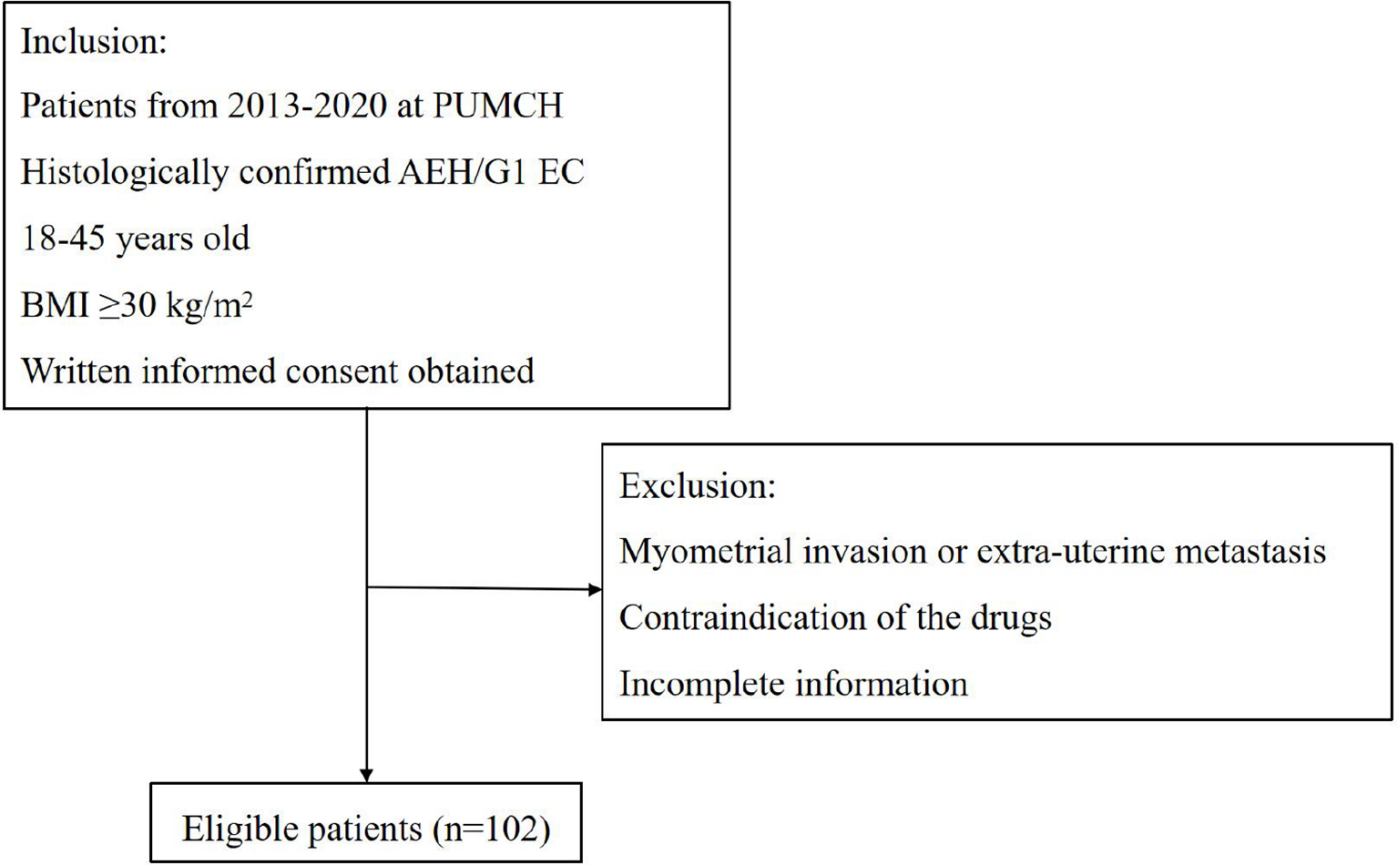
The flowchart of patients’ selection.

All patients were included at the Department of Obstetrics and Gynecology, Peking Union Medical College Hospital (PUMCH) from January 2013 to December 2020. The inclusion criteria were as follows: (1) Histologically confirmed AEH or grade 1 endometrioid adenocarcinoma; (2) women between 18 and 45 years old who desire to preserve their fertility; (3) BMI ≥30 kg/m^2^; (4) no signs of myometrial invasion or extra-uterine metastasis by enhanced magnetic resonance imaging (MRI); (5) no contraindication of the drugs or pregnancy. At the time of diagnosis, all pathology slides were reviewed by pathologists who specialized in gynecologic oncology at our institution. This study was approved by the PUMCH Ethics Committee. **Figure 1** shows the flowchart illustrating patients’ selection.

### Treatment Methods

The treatment protocol was described in our previous work (12). Two regimens were used: (1) Progestin therapy: oral medroxyprogesterone acetate (MPA) 500 mg daily or megestrol acetate (MA) 160 mg bid; (2) gonadotropin-releasing hormone agonist (GnRHa)-based therapy: a combination of subcutaneous 3.75 mg GnRHa injection every 4 weeks and levonorgestrel-releasing intrauterine system (LNG-IUS) (Mirena) insertion constantly or oral letrozole 2.5 mg daily. Weight loss plans including diet control and exercise recommendation were provided to all patients during the whole treatment process. Patients were asked to come to the clinic every 3–4 months for a follow-up evaluation. Physical examination, including body weight, BMI, and body fat detection, and lab tests, including complete blood counts and liver function test, were performed. A transvaginal ultrasound scan was performed at each visit to assess the endometrium. Side effects such as vaginal spotting and abdominal pain were also recorded. During each follow-up, the treatment efficacy was evaluated by endometrial curettage under hysteroscopy.

### Response Evaluation

The treatment outcomes were categorized as complete response (CR), partial response (PR), stable disease (SD), and progressive disease (PD), as illustrated in our previous work (12). CR was defined as the absence of disease. PR indicated histological regression. SD was defined as disease persistence, while PD referred to disease progression to a higher grade or progressive disease. Additional 1–2 treatment courses were performed on patients with PR or SD, whereas those with PD were immediately proposed to undergo a hysterectomy. Patients with the persistent or worsening disease over 12 months were considered failing to respond to therapy and recommended to undergo surgery subsequently. Once CR was achieved, women were encouraged to conceive naturally or referred to undergo assisted reproductive technology (ART) immediately. Patients who had no birth plan were temporarily prescribed to receive maintenance therapy including oral contraceptives, low-dose cyclic progestin, or LNG-IUS insertion to prevent a recurrence.

### Follow-Up

The follow-up schedule was the same as reported in our previous article (12). All patients were regularly followed up for a prolonged period with 3- to 6-month intervals. Information about recurrence and fertility outcomes was documented. If the patient underwent hysterectomy, the reason, post-operative pathology results, and adjuvant therapy were also collected.

### Statistical Analyses

Statistical Package for Social Sciences for Windows (version 22.0) was used for statistical analysis. Data are presented as median values with ranges or as counts with percentages. Chi-squared or Fisher’s exact tests were used for frequency distribution comparison, and median values were compared using Mann–Whitney *U* tests. Possible factors associated with CR and recurrence were investigated with univariate and multivariate analyses using logistic regression, and odds ratios were calculated along with 95% confidence intervals (95% CI). *p*-values < 0.05 were considered statistically significant.

## Results

### Characteristics of Patients With Obesity

One hundred and two obese patients were included in our study. The clinical characteristics of patients are shown in **Table 1**. Forty (39.2%) patients were diagnosed as AEH and 62 (60.8%) were EC. The median age at diagnosis was 32 (21–42) years. The median BMI of patients was 33.5 (30.1–46.1) and 11 (10.8%) patients’ BMI was over 40. Seventy-nine (77.5%) women were nulliparous, and 43 (42.2%) had comorbidities, including polycystic ovary syndrome, endometriosis, and diabetes mellitus. Forty-one (40.2%) women were treated with progestin regimen and 61 (59.8%) were treated with GnRHa combination therapy.

**Table 1:** Patient’s characteristics.

Characteristics		Total (*n* = 102)
Age (years), median (range)		32 (21–42)
BMI (kg/m^2^), median (range)		33.5 (30.1–46.1)
Histology		
EC		62 (60.8%)
AEH		40 (39.2%)
Comorbidity		
PCOS		26 (25.5%)
Endometriosis		6 (5.9%)
DM		9 (8.8%)
Nulliparity		79 (77.5%)
Regimen		
Progestin		41 (40.2%)
GnRHa		61 (59.8%)

BMI, body mass index; EC, endometrial carcinoma; AEH, atypical endometrial hyperplasia; PCOS, polycystic ovary syndrome; DM, diabetes mellitus.

### Treatment Outcomes

Eighty-eight (86.3%) patients achieved CR with a median CR time of 6 months, ranging from 3 to 12 months. Thirteen (12.7%) patients failed to achieve CR: 7 PR, 5 SD, and 1 PD. Nine of the 13 were transferred from progestin regimen to GnRHa and finally achieved CR after 1–2 courses. The other 3 patients underwent hysterectomy. Based on post-operative histological findings, one case was diagnosed as AEH, and the other 2 cases were stage IA EC and 1 combined with stage IC ovarian endometrial carcinoma. The rest of the patients were still in treatment at the final contact.

The CR rate and time in AEH patients were 92.5% and 6 months (3–10 months), respectively. In EC patients, CR rate and time were 82.3% and 7 months (3–12 months), respectively (*p* = 0.142). Univariate analysis indicated that the CR rate was higher in patients who received the GnRHa-based regimen (93.4% vs. 75.6%, *p* = 0.011). High remission rates were also found in patients younger than 35 years old (87.2% vs. 83.3%, *p* = 0.632) and who lost more than 10% of their weight (96.4% vs. 82.4%, *p* = 0.067), and even no significance was found (**Tables 2**, **3**). There was a corresponding increase in the proportion of CR with increased weight loss (**Table 4**).

**Table 2:** Predictors of complete response.

Predictors of complete response	Univariate analysis OR (95% CI)	*p*-value	Multivariate analysis OR (95% CI)	*p*-value
Age: <35 years vs. ≥35 years	1.046 (0.858–1.275)	0.632		
PCOS: no vs. yes	1.022 (0.843–1.239)	0.817		
AEH vs. EC	1.125 (0.972–1.301)	0.142		
Weight loss: <10% vs. ≥ 10%	0.855 (0.753–0.971)	0.067		
Regimen: progestin vs. GnRHa	0.809 (0.672–0.975)	**0.011**	0.232 (0.051–1.052)	0.058

EC, endometrial carcinoma; AEH, atypical endometrial hyperplasia; PCOS, polycystic ovary syndrome. The bold value highlights the p value <0.05.

**Table 3:** Outcome of progestin and GnRHa treatment.

Characteristics	Progestin			GnRHa		
	EC (*n* = 22)	AEH (*n* = 19)	Total (*n* = 41)	EC (*n* = 40)	AEH (*n* = 21)	Total (*n* = 61)
CR						
CR rate	15 (68.1%)	16 (84.2%)	31 (75.6%)	37 (92.5%)	20 (95.2%)	57 (93.4%)
CR time, month (range)	9 (3–12)	6 (3–10)	7 (3–12)	6 (3–12)	5 (3–10)	6 (3–12)
Follow-up time, month (range)	31 (5–83)	30 (3–84)	32 (3–84)	31 (3–92)	28 (3–82)	29 (3–92)
Recurrence						
Recurrence rate	5 (33.3%)	3 (18.7%)	8 (25.8%)	5 (13.5%)	2 (10.0%)	7 (12.2%)
Recurrence time, month (range)	20 (8–52)	33 (30–40)	31 (8–52)	22 (12–36)	28 (26–30)	25 (12–36)
Attempts to conceive	7	9	16	27	14	41
Live birth rate	1 (14.3%)	2 (22.2%)	3 (18.8%)	2 (7.4%)	2 (14.2%)	4 (9.5%)
Miscarriage	2 (28.6%)	0	2 (12.5%)	3 (11.1%)	1 (7.1%)	3 (9.5%)
In pregnancy	0	1 (11.1%)	1 (6.3%)	2 (7.4%)	1 (7.1%)	3 (7.1%)
Total pregnancy rate	3 (42.8%)	3 (33.3%)	6 (37.5%)	7 (25.9%)	4 (28.6%)	11 (26.8%)

CR, complete response; EC, endometrial carcinoma, AEH, atypical endometrial hyperplasia.

**Table 4:** Correlation between body weight change and treatment outcomes.

Outcome	Weight loss		
	<0% (*n* = 44)	0%–10% (*n* = 30)	>10% (*n* = 28)
CR			
CR rate	34 (77.2%)	27(90.0%)	27 (96.4%)
CR time, month (range)	7 (3–12)	6 (3–11)	7 (3–12)
Recurrence			
Recurrence rate	7 (20.5%)	4 (14.8%)	4 (14.8%)
Recurrence time, month (range)	26 (8–40)	37 (12–48)	30 (21–52)
Attempts to conceive	21	18	18
Live birth rate	2 (9.5%)	3 (16.7%)	2 (11.1%)
Miscarriage	2 (9.5%)	1 (11.1%)	2 (11.1%)
In pregnancy	1 (4.8%)	1 (11.1%)	2 (11.1%)
Total pregnancy rate	5 (23.8%)	5 (27.8%)	6 (33.3%)

CR, complete response; EC, endometrial carcinoma; AEH, atypical endometrial hyperplasia.

### Adverse Effects

In patients who received progestin regimen, weight gain was the most common side effect (43.9%), and 26.8% of patients gained weight more than 5%, followed by irregular vaginal bleeding (10.7%) and abnormal liver function (3.9%). In patients who received the GnRHa regimen, postmenopausal symptoms such as hot flashes and vaginal dryness were the most common adverse reactions (19.8%). The degree of these symptoms was minor and no patients received add-back estrogen. Irregular bleeding was also observed in 11.5% of the patients, but no weight gain, liver dysfunction, and IUD dislocation were recorded. In all patients, no major complications or adverse effects required suspension of treatment. No treatment-related deaths were identified.

### Recurrence

After a pathological CR was achieved, 66 patients accepted maintenance treatment, including LNG-IUS, cyclical oral contraceptives, or low-dose cyclic progestin until they began attempting gestation. Twenty-two patients were only followed up regularly without any treatment. After a median follow-up time of 31 months (3–92 months), 15 (17.0%) women developed recurrence with a 26-month median recurrence time, ranging from 8 to 52 months. Seven patients who gave up to preserve their uterus chose to receive hysterectomy with or without lymphadenectomy. Eight patients received fertility-sparing re-treatment after recurrence, and 5 (62.5%) of them achieved CR again. Two (25%) of them underwent hysterectomy due to SD, and both of them were diagnosed as stage IA EC based on post-operative histology. The last patient was still in treatment at the final contact. No patient died due to the disease during this period.

The recurrence-related factors are shown in **Table 5**. The recurrence rate was 13.9% in AEH and 19.2% in EC (*p* = 0.691). Patients who received the GnRHa regimen, lost more than 10% weight, received maintenance therapy, or conceived during the follow-up period had a low probability of recurrence. Also, the recurrence rate decreased more, with more weight patients lost (**Table 4**).

**Table 5:** Predictors of recurrence.

Risk factors to recurrence	Univariate analysis HR (95% CI)	*p*-value
Age: <35 years vs. ≥35 years	1.176 (0.368–3.263)	0.782
PCOS: no vs. yes	0.750 (0.286–1.970)	0.563
AEH vs. EC	0.962 (0.791–1.169)	0.691
Weight loss: <10% vs. ≥10%	1.217 (0.426–3.817)	0.711
Regimen: progestin vs. GnRHa	2.101 (0.841–5.248)	0.107
Maintenance therapy: no vs. yes	1.941 (0.765–4.929)	0.173
Conceive: no vs. yes	1.444 (0.361–5.780)	0.593

EC, endometrial carcinoma; AEH, atypical endometrial hyperplasia; PCOS, polycystic ovary syndrome.

### Fertility Outcomes

Fifty-seven women attempted to conceive after achieving CR, and 28 (49.1%) were transferred to receive ART. In total, 16 (28.1%) women became pregnant, 7 (12.3%) of them successfully delivered, and 4 (7.0%) were in pregnancy, while 5 (8.8%) of them miscarried, 4 at the first trimester and 1 at the second trimester. The pregnancy rate was superior in patients younger than 35 years old (34.0% vs. 0%, *p* = 0.031). Higher probability was also observed in patients with AEH (30.4% vs. 26.5%, *p* = 0.744), who received progestin therapy (37.5% vs. 24.4%, *p* = 0.322), who lost more than 10% weight (33.3% vs. 25,6%, *p* = 0.548), and who received ART (35.7% vs. 20.7%, *p* = 0.207). Also, the more weight patients lost, the higher tendency of pregnancy was observed (**Table 4**).

## Discussion

As the major risk factor for EC, obesity has become a global public health problem (13, 14). According to the World Health Organization, obesity is defined as BMI ≥30 kg/m^2^ (15). Women with obesity have about a 2.6- to 4.7-fold increase in the risk for EC compared with women of normal weight (16). With the number of obese populations increasing significantly, the proportion of EC in patients of childbearing age has increased accordingly. Fertility-sparing treatments have been applied to young women with EC/AEH to preserve their uterus with a high remission rate (17–22). Conservative therapy such as progestin therapy is widely accepted as the main method with satisfactory results. Also, it is possible to manage young patients using not only medical therapy but also hysteroscopic resection (23). However, progestin therapy is associated with a significant increase in body weight (3–5). For obese patients, further weight gain may lead to a higher risk of treatment failure, a high relapse rate, and a low possibility of live birth. Therefore, it is a big challenge for us to manage patients with obesity and select the most effective methods for these obese women. However, only a few studies have reported the outcomes of conservative management for obese patients or only as part of their report until now (24, 25). The effect of weight change during treatment on oncologic and reproductive outcomes are yet to be well assessed. This study aimed to investigate the oncological and reproductive outcomes of fertility-preserving treatment for EC and AEH patients with BMI ≥30. We also compared the efficacy and safety of different regimens as well as evaluated the influence of weight change during treatment on CR, recurrence, and pregnancy.

In our cohort, over 90% AEH and 80% EC patients achieved CR with 6 months of median CR time, proving that fertility preservation was feasible for obese women. Both GnRHa and progestin regimens showed great therapeutic effects, whereas a higher remission rate was found in GnRHa-based regimen with CR rate up to 93.4%. In previous studies for fertility-sparing progestin therapy, overweight or obesity was significantly associated with a poor response to progestin therapy (26, 27), this is consistent with our results. In our research, progestin therapy was associated with a significant increase in body weight. Although weight control was recommended for all patients, about 43.9% of the patients gained weight, and 26.8% gained more than 5% of their weight. Among the patients who received progestin therapy but failed to achieve CR, 9 women were transferred to receive GnRHa based therapy and finally got complete remission after 1–2 courses. Our previous studies proved that GnRHa combination treatment in patients who failed oral progestin therapy produced good treatment and reproductive outcomes (28). Also, a low recurrence rate was found in GnRHa-based therapy compared with progestin.

Consequently, progesterone alone therapy may not be the most efficient treatment regimen, and the GnRHa combination regimen could be an alternative for obese patients. Nevertheless, the long-term adverse effect of GnRHa and its influence on fertility by repeated curettage need to be noted owing to the low pregnancy rate of the GnRHa regimen. It has been documented that 2–3% of bone mass will be lost with 6 months of GnRHa use. In addition, it is unclear what is the maximal duration of GnRHa therapy, whether the add-back therapy should be performed, whether the bone mineral density should be monitored, and whether calcium and bisphosphonates should be added. Therefore, the long-term side effect of GnRHa such as osteoporosis and cardiovascular complications should be considered in future studies.

Weight loss is crucial in the clinical management of young patients undergoing fertility-sparing therapy. A previous study suggested that weight change has little influence on complete response and recurrence rates during treatment, while obesity was a significant predictor for low response rates and high recurrence rates (25). However, the correlation between treatment outcome and weight change has rarely been mentioned, especially for obese patients. In this study, obese patients who lost less than 10% of their weight had a poor response and pregnancy outcomes, as well as a higher rate of recurrence, consistent with previous studies (6). Besides, the more weight patients lost, the higher the CR rate observed. Thus, weight control and health consulting are crucial in the whole-lifespan management of fertility-sparing treatment. GnRHa-based therapy has an advantage on weight control since it is well known that weight gain is the main side effect of high-dose oral progestin. Further research concerning the correlation between weight loss and pregnancy loss is still needed.

The Cancer Genome Atlas (TCGA)-based molecular classification system and MMR testing have been proposed in young women desiring fertility-sparing treatment, but the association is still unclear (29). Some researches revealed a 100% recurrence rate in MMR-deficient patients and suggested that patients with Lynch syndrome and P53 mutations should not be treated conservatively (30, 31). Owing to data limitations in our study, we failed to conduct further research in this aspect, Still, we believe that the TCGA classification system can contribute to selecting populations who suit fertility-sparing treatment and help to predict the oncologic outcomes (32, 33).

Previous studies revealed a high rate of relapse of fertility-sparing treatment in EC patients (17, 18). In our research, about 20% of women had developed recurrence with 26 months of median recurrence time, in accordance with former research. However, some of the recurrences occurred as early as 8 months after a CR. Another study reported that some recurrence occurred at 3–4 months after CR, which mandates the follow-up to be started early (34). The longest recurrence in our research took place at 52 months, and recurrence occurring at 13 years was also reported in other previous studies (35, 36). Therefore, long-term monitoring and regular follow-up are essential. Additionally, hormonal maintenance therapy is crucial for women who have no birth plan immediately after completion of treatment (34, 37). Moreover, a low recurrence rate was also found in patients with pregnancy. Herein, maintenance therapy and conception immediately were encouraged to reduce the risk of recurrence. For recurrent patients, fertility-sparing re-treatment could be considered after complete evaluation. Over 60% of recurrent patients achieved CR again in our research. In our previous study, the CR rate was about 90% in BMI normal patients. However, with increased treatment times, the recurrence time shortened, and no patients got pregnant after the third-round treatment (12). Patients were supposed to be informed of treatment failure and conceive before the re-treatment. Given the limited number of patients and several patients still being treated, whose therapeutic efficacy is yet to be assessed, we assumed that a future study with a larger samples size should be performed to evaluate the effect.

Conception is the ultimate goal of uterine preservation for most patients. However, the pregnancy and live birth rates in our research are still somewhat suboptimal, lower than previous studies (27, 38). However, the miscarriage rate at the first or second trimester is in accordance with the ordinary population (39). This might be due to patients included in our studies being obese women, which was associated with a lower probability of pregnancy. Moreover, the follow-up time in our study was relatively short; if longer follow-up times were performed, a high rate of relapse and live birth might be observed (40). The correlation between weight loss and reproductive outcomes has not been well evaluated and reported before. Some studies believed that the weight loss was not related to pregnancy and live birth rates in EC patients who received fertility-preserving treatment. In contrast, our current results showed that patients who lost weight more than 10% had a higher pregnancy rate. So, we concluded that weight loss could positively affect the pregnancy and live birth rate in obese women. It has been reported that weight loss ≥5% could also improve the pregnancy rate as an independent positive factor (41). Therefore, weight loss or recovery patients’ normal BMIs during therapy as well as the ART time is of great significance. Despite our expectations, ART did not significantly improve the live birth rate, women who chose IVF-ET had relatively better results, and some studies did report improved birth rate with ART (42, 43). Hence, once CR has been achieved, getting pregnant should be considered as soon as possible, and IVF-ET is recommended.

## Limitations

Firstly, our study was a single-center retrospective study, and the choice of individual regimens was essentially a matter of physician preference. Multi-center prospective clinical trials are supposed to be conducted to verify the efficacy. Secondly, some patients were still under treatment until the last contact, which may influence the research results. Thirdly, the follow-up time of our study is relatively limited, and long-term follow-up was recommended to verify high pregnancy and recurrence rates.

## Conclusions

In conclusion, the findings of our study confirm that fertility-sparing treatment for obese women with EC/AEH appears to be an acceptable method. The combination of GnRHa with LNG-IUS/letrozole is an alternative regimen with a higher regression rate and low rate of recurrence, as well as fewer side effects such as weight gain compared with progestin. Besides, weight loss of more than 10% positively influences CR, recurrence, and pregnancy rates. Therefore, weight control and health consulting are crucial in the whole-lifespan management of fertility-sparing treatment, especially for obese patients.

## Data Availability Statement

The raw data supporting the conclusions of this article will be made available by the authors, without undue reservation.

## Author Contributions

Conceptualization: JC and DC. Data curation: JC, DC, JY, MY, HZ, JW, YZ, NC, PP, and KS. Formal analysis: JC and DC. Software: JC. Writing—original draft: JC and DC. Writing—review and editing: JY, MY, HZ, JW, YZ, NC, PP, and KS. All authors contributed to the article and approved the submitted version.

## Funding

This work was supported by the Non-profit Central Research Institute Fund of Chinese Academy of Medical Sciences (2020-PT320-003).

## Conflict of Interest

The authors declare that the research was conducted in the absence of any commercial or financial relationships that could be construed as a potential conflict of interest.

## Publisher’s Note

All claims expressed in this article are solely those of the authors and do not necessarily represent those of their affiliated organizations, or those of the publisher, the editors and the reviewers. Any product that may be evaluated in this article, or claim that may be made by its manufacturer, is not guaranteed or endorsed by the publisher.

## References

[1] Lu KH Broaddus RR. Endometrial Cancer. N Engl J Med (2020) 383(21):2053–64. doi: 10.1056/NEJMra1514010 33207095

[2] Li M Guo T Cui R Feng Y Bai H Zhang Z. Weight Control Is Vital for Patients With Early-Stage Endometrial Cancer or Complex Atypical Hyperplasia Who Have Received Progestin Therapy to Spare Fertility: A Systematic Review and Meta-Analysis. Cancer Manag Res (2019) 11:4005–21. doi: 10.2147/CMAR.S194607 PMC6512613 31190979

[3] Gunderson CC Fader AN Carson KA Bristow RE. Oncologic and Reproductive Outcomes With Progestin Therapy in Women With Endometrial Hyperplasia and Grade 1 Adenocarcinoma: A Systematic Review. Gynecol Oncol (2012) 125(2):477–82. doi: 10.1016/j.ygyno.2012.01.003 22245711

[4] Guillon S Popescu N Phelippeau J Koskas M. A Systematic Review and Meta-Analysis of Prognostic Factors for Remission in Fertility-Sparing Management of Endometrial Atypical Hyperplasia and Adenocarcinoma. Int J Gynaecol Obstet (2019) 146(3):277–88. doi: 10.1002/ijgo.12882 31197826

[5] Yu M Yang JX Wu M Lang JH Huo Z Shen K. Fertility-Preserving Treatment in Young Women With Well-Differentiated Endometrial Carcinoma and Severe Atypical Hyperplasia of Endometrium. Fertil Steril (2009) 92(6):2122–4. doi: 10.1016/j.fertnstert.2009.06.013 19591991

[6] Shafiee MN Chapman C Barrett D Abu J Atiomo W. Reviewing the Molecular Mechanisms Which Increase Endometrial Cancer (EC) Risk in Women With Polycystic Ovarian Syndrome (PCOS): Time for Paradigm Shift? Gynecol Oncol (2013) 131(2):489–92. doi: 10.1016/j.ygyno.2013.06.032 23822891

[7] Aubrey C Black K Campbell S Pin S. Endometrial Cancer and Bariatric Surgery: A Scoping Review. Surg Obes Relat Dis (2019) 15(3):497–501. doi: 10.1016/j.soard.2018.12.003 30700395

[8] Calle EE Rodriguez C Walker-Thurmond K Thun MJ. Overweight, Obesity, and Mortality From Cancer in a Prospectively Studied Cohort of U.S. Adults. N Engl J Med (2003) 348(17):1625–38. doi: 10.1056/NEJMoa021423 12711737

[9] von Gruenigen VE Tian C Frasure H Waggoner S Keys H Barakat RR. Treatment Effects, Disease Recurrence, and Survival in Obese Women With Early Endometrial Carcinoma: A Gynecologic Oncology Group Study. Cancer (2006) 107(12):2786–91. doi: 10.1002/cncr.22351 17096437

[10] Bjørge T Häggström C Ghaderi S Nagel G Manjer J Tretli S. BMI and Weight Changes and Risk of Obesity-Related Cancers: A Pooled European Cohort Study. Int J Epidemiol (2019) 48(6):1872–85. doi: 10.1093/ije/dyz188 31566221

[11] Onstad MA Schmandt RE Lu KH. Addressing the Role of Obesity in Endometrial Cancer Risk, Prevention, and Treatment. J Clin Oncol (2016) 34(35):4225–30. doi: 10.1200/JCO.2016.69.4638 PMC5455320 27903150

[12] Chen J Cao D Yang J Yu M Zhou H Cheng N. Management of Recurrent Endometrial Cancer or Atypical Endometrial Hyperplasia Patients After Primary Fertility-Sparing Therapy. Front Oncol (2021) 11:738370. doi: 10.3389/fonc.2021.738370 34568074 PMC8458864

[13] Kiesel L Eichbaum C Baumeier A Eichbaum M. Obesity Epidemic-The Underestimated Risk of Endometrial Cancer. Cancers (Basel) (2020) 12(12):3860. doi: 10.3390/cancers12123860 PMC7767192 33371216

[14] Reeves GK Pirie K Beral V Green J Spencer E Bull D. Cancer Incidence and Mortality in Relation to Body Mass Index in the Million Women Study: Cohort Study. Bmj (2007) 335(7630):1134. doi: 10.1136/bmj.39367.495995.AE 17986716 PMC2099519

[15] Januszek SM Barnas E Skret-Magierlo J Sokolowski J Sczerba P Januszek R. Obesity as a Risk Factor of in-Hospital Outcomes in Patients With Endometrial Cancer Treated With Traditional Surgical Mode. Ginekol Pol (2019) 90(10):549–56. doi: 10.5603/GP.2019.0095 31686410

[16] Shaw E Farris M McNeil J Friedenreich C. Obesity and Endometrial Cancer. Recent Results Cancer Res (2016) 208:107–36. doi: 10.1007/978-3-319-42542-9_7 27909905

[17] Leone Roberti Maggiore U Martinelli F Dondi G Bogani G Chiappa V Evangelista MT. Efficacy and Fertility Outcomes of Levonorgestrel-Releasing Intra-Uterine System Treatment for Patients With Atypical Complex Hyperplasia or Endometrial Cancer: A Retrospective Study. J Gynecol Oncol (2019) 30(4):e57. doi: 10.3802/jgo.2019.30.e57 31074240 PMC6543108

[18] Yamagami W Susumu N Makabe T Sakai K Nomura H Kataoka F. Is Repeated High-Dose Medroxyprogesterone Acetate (MPA) Therapy Permissible for Patients With Early Stage Endometrial Cancer or Atypical Endometrial Hyperplasia Who Desire Preserving Fertility? J Gynecol Oncol (2018) 29(2):e21. doi: 10.3802/jgo.2018.29.e21 29400014 PMC5823982

[19] Tamauchi S Kajiyama H Utsumi F Suzuki S Niimi K Sakata J. Efficacy of Medroxyprogesterone Acetate Treatment and Retreatment for Atypical Endometrial Hyperplasia and Endometrial Cancer. J Obstet Gynaecol Res (2018) 44(1):151–6. doi: 10.1111/jog.13473 29121428

[20] Park JY Kim DY Kim JH Kim YM Kim KR Kim YT. Long-Term Oncologic Outcomes After Fertility-Sparing Management Using Oral Progestin for Young Women With Endometrial Cancer (KGOG 2002). Eur J Cancer (2013) 49(4):868–74. doi: 10.1016/j.ejca.2012.09.017 23072814

[21] Giampaolino P Di Spiezio Sardo A Mollo A Raffone A Travaglino A Boccellino A. Hysteroscopic Endometrial Focal Resection Followed by Levonorgestrel Intrauterine Device Insertion as a Fertility-Sparing Treatment of Atypical Endometrial Hyperplasia and Early Endometrial Cancer: A Retrospective Study. J Minim Invasive Gynecol (2019) 26(4):648–56. doi: 10.1016/j.jmig.2018.07.001 30017893

[22] Falcone F Laurelli G Losito S Di Napoli M Granata V Greggi S. Fertility Preserving Treatment With Hysteroscopic Resection Followed by Progestin Therapy in Young Women With Early Endometrial Cancer. J Gynecol Oncol (2017) 28(1):e2. doi: 10.3802/jgo.2017.28.e2 27670256 PMC5165067

[23] Casadio P La Rosa M Alletto A Magnarelli G Arena A Fontana E. Fertility Sparing Treatment of Endometrial Cancer With and Without Initial Infiltration of Myometrium: A Single Center Experience. Cancers (Basel) (2020) 12(12):3571. doi: 10.3390/cancers12123571 PMC7760930 33260382

[24] Cholakian D Hacker K Fader AN Gehrig PA Tanner EJ 3rd. Effect of Oral Versus Intrauterine Progestins on Weight in Women Undergoing Fertility Preserving Therapy for Complex Atypical Hyperplasia or Endometrial Cancer. Gynecol Oncol (2016) 140(2):234–8. doi: 10.1016/j.ygyno.2015.12.010 26706662

[25] Park JY Seong SJ Kim TJ Kim JW Bae DS Nam JH. Significance of Body Weight Change During Fertility-Sparing Progestin Therapy in Young Women With Early Endometrial Cancer. Gynecol Oncol (2017) 146(1):39–43. doi: 10.1016/j.ygyno.2017.05.002 28526167

[26] Penner KR Dorigo O Aoyama C Ostrzega N Balzer BL Rao J. Predictors of Resolution of Complex Atypical Hyperplasia or Grade 1 Endometrial Adenocarcinoma in Premenopausal Women Treated With Progestin Therapy. Gynecol Oncol (2012) 124(3):542–8. doi: 10.1016/j.ygyno.2011.11.004 22079678

[27] Chen M Jin Y Li Y Bi Y Shan Y Pan L. Oncologic and Reproductive Outcomes After Fertility-Sparing Management With Oral Progestin for Women With Complex Endometrial Hyperplasia and Endometrial Cancer. Int J Gynaecol Obstet (2016) 132(1):34–8. doi: 10.1016/j.ijgo.2015.06.046 26493012

[28] Chen JY Cao DY Zhou HM Yu M Yang JX Wang JH. GnRH-A Combined Fertility-Sparing Re-Treatment in Women With Endometrial Carcinoma or Atypical Endomertial Hyperplasia Who Failed to Oral Progestin Therapy. Zhonghua Fu Chan Ke Za Zhi (2021) 56(8):561–8. doi: 10.3760/cma.j.cn112141-20210603-00298 34420288

[29] Soslow RA Tornos C Park KJ Malpica A Matias-Guiu X Oliva E. Endometrial Carcinoma Diagnosis: Use of FIGO Grading and Genomic Subcategories in Clinical Practice: Recommendations of the International Society of Gynecological Pathologists. Int J Gynecol Pathol (2019) 38 Suppl 1(Iss 1 Suppl 1):S64–74. doi: 10.1097/PGP.0000000000000518 30550484 PMC6295928

[30] Raffone A Catena U Travaglino A Masciullo V Spadola S Della Corte L. Mismatch Repair-Deficiency Specifically Predicts Recurrence of Atypical Endometrial Hyperplasia and Early Endometrial Carcinoma After Conservative Treatment: A Multi-Center Study. Gynecol Oncol (2021) 161(3):795–801. doi: 10.1016/j.ygyno.2021.03.029 33812697

[31] Britton H Huang L Lum A Leung S Shum K Kale M. Molecular Classification Defines Outcomes and Opportunities in Young Women With Endometrial Carcinoma. Gynecol Oncol (2019) 153(3):487–95. doi: 10.1016/j.ygyno.2019.03.098 30922603

[32] Raffone A Travaglino A Raimondo D Boccellino MP Maletta M Borghese G. Tumor-Infiltrating Lymphocytes and POLE Mutation in Endometrial Carcinoma. Gynecol Oncol (2021) 161(2):621–8. doi: 10.1016/j.ygyno.2021.02.030 33715893

[33] Raffone A Travaglino A Raimondo D Neola D Renzulli F Santoro A. Prognostic Value of Myometrial Invasion and TCGA Groups of Endometrial Carcinoma. Gynecol Oncol (2021) 162(2):401–6. doi: 10.1016/j.ygyno.2021.05.029 34088515

[34] Novikova OV Nosov VB Panov VA Novikova EG Krasnopolskaya KV Andreeva YY. Live Births and Maintenance With Levonorgestrel IUD Improve Disease-Free Survival After Fertility-Sparing Treatment of Atypical Hyperplasia and Early Endometrial Cancer. Gynecol Oncol (2021) 161(1):152–9. doi: 10.1016/j.ygyno.2021.01.001 33461741

[35] Fujiwara H Jobo T Takei Y Saga Y Imai M Arai T. Fertility-Sparing Treatment Using Medroxyprogesterone Acetate for Endometrial Carcinoma. Oncol Lett (2012) 3(5):1002–6. doi: 10.3892/ol.2012.602 PMC3389624 22783380

[36] Wang CJ Chao A Yang LY Hsueh S Huang YT Chou HH. Fertility-Preserving Treatment in Young Women With Endometrial Adenocarcinoma: A Long-Term Cohort Study. Int J Gynecol Cancer (2014) 24(4):718–28. doi: 10.1097/IGC.0000000000000098 24577149

[37] Wang Y Zhou R Wang H Liu H Wang J. Impact of Treatment Duration in Fertility-Preserving Management of Endometrial Cancer or Atypical Endometrial Hyperplasia. Int J Gynecol Cancer (2019) 29(4):699–704. doi: 10.1136/ijgc-2018-000081 30826750

[38] Chae SH Shim SH Lee SJ Lee JY Kim SN Kang SB. Pregnancy and Oncologic Outcomes After Fertility-Sparing Management for Early Stage Endometrioid Endometrial Cancer. Int J Gynecol Cancer (2019) 29(1):77–85. doi: 10.1136/ijgc-2018-000036 30640687

[39] Ammon Avalos L Galindo C Li DK. A Systematic Review to Calculate Background Miscarriage Rates Using Life Table Analysis. Birth Defects Res A Clin Mol Teratol (2012) 94(6):417–23. doi: 10.1002/bdra.23014 22511535

[40] Gallos ID Gupta JK. Comment on: What About the Relapse of Endometrial Hyperplasia? Eur J Obstet Gynecol Reprod Biol (2013) 171(2):e5. doi: 10.1016/j.ejogrb.2011.09.026 21992962

[41] Zhang Y Li D Yan Q Song X Tian W Wang Y. Weight Loss Improves Pregnancy and Livebirth Outcomes in Young Women With Early-Stage Endometrial Cancer and Atypical Hyperplasia. Cancer Manag Res (2021) 13:5711–22. doi: 10.2147/CMAR.S316040 PMC8286730 34285588

[42] Hertz-Picciotto I Samuels SJ. Incidence of Early Loss of Pregnancy. N Engl J Med (1988) 319(22):1483–4. doi: 10.1056/NEJM198812013192214 3185669

[43] Park JY Seong SJ Kim TJ Kim JW Kim SM Bae DS. Pregnancy Outcomes After Fertility-Sparing Management in Young Women With Early Endometrial Cancer. Obstet Gynecol (2013) 121(1):136–42. doi: 10.1097/AOG.0b013e31827a0643 23262938

